# Supercurrent Induced by Chiral Coupling in Multiferroic/Superconductor Nanostructures

**DOI:** 10.3390/nano11010184

**Published:** 2021-01-13

**Authors:** Bjoern Niedzielski, Chenglong Jia, Jamal Berakdar

**Affiliations:** 1 Institut für Physik, Martin-Luther Universität Halle-Wittenberg, 06099 Halle (Saale), Germany; bjoern.niedzielski@student.uni-halle.de; 2 Key Laboratory for Magnetism and Magnetic Materials of the Ministry of Education and Institute of Theoretical Physics, Lanzhou University, Lanzhou 730000, China; cljia@lzu.edu.cn

**Keywords:** superconductor, multiferroic/superconductor nanostructures, superconducting vortices

## Abstract

We study the transport and the superconducting dynamics in a layer of type II superconductor (SC) with a normal top layer that hosts a helical magnetic ordering that gives rise to spin-current-driven ferroelectric polarization. Proximity effects akin to this heterostructure result in an anisotropic supercurrent transport and modify the dynamic properties of vortices in the SC. The vortices can be acted upon and controlled by electric gating or other means that couple to the spin ordering in the top layer, which, in turn, alter the superconducting/helical magnet coupling characteristics. We demonstrate, using the time dependent Ginzburg–Landau approach, how the spin helicity of the top layer can be utilized for pinning and guiding the vortices in the superconducting layer.

## 1. Introduction

Based on the Ginzburg–Landau (GL) theory for superconductors, A.A. Abrikosov predicted two distinct classes of superconductors depending on the parameter κ, which quantifies the ratio between the magnetic field penetration depth $\lambda_{GL}$ and the coherence length $\xi_{GL}$ of the superconducting state. In type-II superconductors with $\kappa >1/\sqrt{2}$, the magnetic flux of an applied magnetic field is no longer expelled completely and it penetrates the material in the form of filaments of quantized flux. These filaments, called vortices or fluxons, are whirls of supercurrent around a normal conducting core, and for mesoscopic systems they form a lattice due to their mutual repulsion. From a technological point of view it is crucial to understand how the vortex lattice behaves under the application of a transport current, since moving fluxons cause a finite electrical resistance and energy dissipation in SCs. Much effort has been made to find effective ways to pin vortices and thereby enhance the critical current of the SC. A class of methods utilizes artificial pinning centers such as nanoparticles, antidots, columnar defects, thickness modulations, and magnetic dots on top of the SC [1,2,3,4,5]. In addition, the pinning potential of magnetic stray fields in coupled ferromagnet/superconductor (FM/SC) multilayers has been explored. Here, the idea is to pin the magnetic flux of the vortex rather than its normal conducting core by attaching the SC to a strong-anisotropy FM [6]. For magnetic fields below the coercive field, the stray fields of the domains in the FM are able to pin vortices—an effect that has been predicted to be potentially much stronger than pinning by conventional methods [7]. There is a further property of mesoscopic superconductors that can be utilized for vortex control. Microsized SCs of non-elipsoidal shape are known to display large energy barriers for flux penetration and expulsion. In general, these surface barriers depend in a complicated way on the material properties as well as on the geometry of the specimen, which makes their systematic investigation challenging [8,9,10]. Nonetheless, the understanding of them is of great importance for applications. They are not only a source of irreversibility of the superconductor’s magnetic response, but they can also serve to keep fluxons out of the SC, improving the device efficiency. In combination with conventional pinning methods, the exploitation of the penetration barrier can greatly enhance the critical current of an SC [11]. The problem of the surface barrier has been under investigation for several decades and is still of current importance. This problem is also of relevance to our system. Below, we discuss how it affects the flux pinning in the presence of spin–orbit coupling (SOC). Here, we investigate the transport properties and the flux flow in a SC that is proximity-coupled to a helical multiferroic oxide such as ${\mathrm{TbMnO}}_{3}$ [12]. In such noncollinearly magnetically ordered compounds, the interplay of electronic correlation, the exchange field, and the spin–orbit interaction causes a spontaneous (spin-driven) electric polarization, meaning ferroelectricity and magnetism are coupled. The emergence of the ferroelectric polarization thus offers the opportunity to act on the spin ordering via an electric field [13,14]. For helical spin ordering, the switching from positive to negative spin helicity can be achieved by a moderate gate voltage, modifying in turn the characteristics of the coupling to the SC, in particular the SOC. Thus, the multiferroic layer can be utilized as a control element for flux motion in the SC. We show that by altering the helicity of the spin-spiral, the vortex motion undergoes subsequent phases of viscous flow and pinning. An inversion of the helicity can transit the system from the resistive to the dissipationless state. The mechanism that couples the spin ordering to the superconducting phase field is in our case an interfacial spin–orbit coupling caused by the proximity to the oxide layer on top of the SC. We find that the SOC-induced supercurrents modify the edge barrier for vortex entry into the system. We identify this barrier modification as the key factor that drives flux motion. Furthermore, it is observed that the ferroelectric top layer renders the fluxon transport anisotropic, meaning the transport depends on the mutual orientation of the transport current density $j_{e}$ and the wave vector of the spin-spiral k. For $j_{e}\|k$ the vortex motion can be strongly inhibited depending on |k|, whereas for $j_{e}⊥k$ the vortex motion becomes channeled.

## 2. Methods

**Figure 1:**
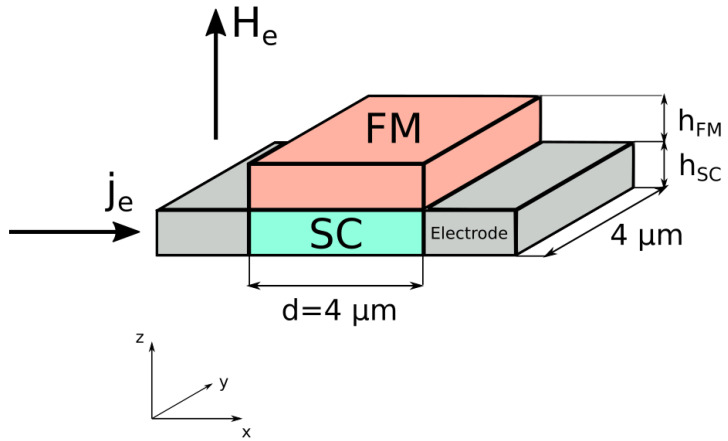
Schematic of the system. A superconducting square film of length *d* and height $h_{SC}$ placed underneath a square non-superconducting film of height $h_{FM}$ that hosts a helical spin order with a spin-driven ferroelectricity, meaning we are dealing with a multiferroic (MF) layer. The bilayer is subject to an external magnetic field $H_{e}=H_{e}e_{z}$, and the external current $j_{e}=j_{e}e_{x}$ flows in the superconductor (SC).

The system under investigation is a superconducting thin film, which is proximity coupled to a magnetically ordered top layer that hosts a transverse-spiral spin ordering. In the following we will refer to this layer as magnetic, though technically the equilibrium state has no averaged magnetic moment but a finite spin-driven ferroelectric polarization. In the simulations we choose both layers to be square films with a side length of d=4 μm and heights $h_{SC}$ and $h_{FM}$ (see Figure 1). The whole heterostructure is subject to an external magnetic field $B_{e}=B_{e}e_{z}$, and is driven by a transport current $j_{e}$. Two electrodes are attached to the sides where the current is injected into the SC. In the cases considered here we have either $j_{e}=j_{e}e_{x}$ or $j_{e}=j_{e}e_{y}$. Generally, the broken space-inversion symmetry at the interface between the FM and SC allows for a Rashba spin–orbit coupling between the materials. In our case, the magnetic layers host an intrinsic SOC associated with the helical spin ordering as well as the ferroelectricity of the top layer. Thus, a local exchange coupling to the SC results effectively in a magneto–electric interaction between the superconducting phase field and the magnetic texture [15,16,17]. This interaction is manifested in a supercurrent that flows at the interface of the bilayer even in the absence of external magnetic fields. We demonstrate how this current can be utilized to control the dynamics of superconducting vortices.

The starting point for the following analysis is the free energy of the SC in the presence of SOC [18]

(1) $$G=\int_{\Omega_{SC}}\left({\left|\Psi \right|}^{2}\left[a+\frac{b}{2}{\left|\Psi \right|}^{2}\right]+\frac{1}{2m_{s}}{\left|\hat{D}\Psi \right|}^{2}+\frac{\alpha }{2m_{s}}\cdot \left[\Psi {\left(\hat{D}\Psi \right)}^{⋆}+\Psi^{⋆}\left(\hat{D}\Psi \right)\right]+\frac{B^{2}}{2\mu_{0}}-B\cdot H_{e}\right)dV.$$

The coefficients $a=a_{0}\left(T-T_{c}\right)$ and b>0 follow from conventional GL-theory, where $T_{c}$ is the critical temperature of the SC and $a_{0}>0$. The condensate wave function $\Psi =\Psi_{0}e^{i\theta }$ is characterized by its amplitude $\Psi_{0}$ and phase θ. The gauge invariant momentum operator is $\hat{D}=-i\hbar \nabla -q_{s}A$, and the magnetic flux density is B=∇×A. The applied magnetic field is $H_{e}=B_{e}/\mu_{0}$. The constants $m_{s}$ and $q_{s}$ are the mass and charge of a Cooper pair. The term proportional to $\alpha =\alpha_{0}N\times h$ is the free energy density due to Rashba-SOC and is only allowed in the absence of space-inversion symmetry. It enters the free energy density in the form of a Lifshitz invariant [19]. The normal vector N points in the direction of the axis along which the symmetry is broken, in our case this is the *z*-direction. The exchange field h is related to the magnetic texture and penetrates the SC in a thin layer around the interface. Only there is the Rashba parameter $\alpha_{0}$ finite. For thin films with $h_{SC}\ll \lambda_{GL}$, the spin–orbit coupling is averaged out over the complete layer [20,21]. We follow this argument with additionally imposing $h_{SC}\ll \xi_{GL}$. In this way the wave function Ψ is approximately constant over the width of the layer. Minimizing the free energy with respect to $\Psi^{⋆}$ and A yields the well known time-dependent Ginzburg–Landau equations (TDGL)

(2) $$\frac{\delta G}{\delta \Psi^{⋆}}=\gamma \left(\hbar \frac{\partial \Psi }{\partial t}+iq_{s}\phi \Psi \right)$$

(3) $$\frac{\delta G}{\delta A}=-\sigma \left(\frac{\partial A}{\partial t}+\nabla \phi \right).$$

Here $\gamma =\hbar /(2m_{s}D)$ is a dimensionless relaxation coefficient that determines the relaxation time of the system, σ is the electric conductivity of the normal state, $D=v_{f}l/3$ is a phenomenological diffusion constant, and $v_{f}$ and *l* are the Fermi velocity and the electron mean free path, respectively. The electric scalar potential ϕ will be utilized to simulate transport currents through the SC. The electric currents in- and outside the SC are the source of the vector potential A. Specifically we have dissipationless supercurrents $j_{s}$, normal electric currents $j_{n}=-\sigma \left(\partial_{t}A+\nabla \phi \right)$, and current flow $j_{ext}=\nabla \times H_{e}$ in a coil that generates the external magnetic field $B_{e}=B_{e}e_{z}$. For the total vector potential we choose the Coulomb gauge ∇·A=0, whereas the vector potential corresponding to external currents is formulated in the symmetric gauge $A_{e}=0.5B_{e}\left(-ye_{x}+xe_{y}\right)$. The TDGL were originally proposed by Schmid with the aim to find the simplest model that adequately describes nonequilibrium phenomena, like flux flow and relaxation processes in SCs [22]. For more information about the phenomenological TDGL model and its limits, as well as consistency issues also in connection with more microscopic approaches, we refer to the literature [23,24,25,26]. After performing the functional derivatives in Equations (2) and (3), the TDGL take on the following form:

(4) $$0=\gamma \left(\hbar \frac{\partial \Psi }{\partial t}+iq_{s}\phi \Psi \right)+\Psi \left({b|\Psi |}^{2}-\left|a\right|-\frac{\alpha^{2}}{2m_{s}}\right)+\frac{1}{2m_{s}}{\left(\hat{D}+\alpha \right)}^{2}\Psi$$

(5) $$0=-\frac{1}{\mu_{0}}\nabla \times B-\sigma \left(\frac{\partial A}{\partial t}+\nabla \phi \right)+\nabla \times H_{e}+j_{s}$$

(6) $$0=-\sigma \Delta \phi +\nabla \cdot j_{s}.$$

with supercurrent density

(7) $$j_{s}=i\frac{q_{s}\hbar }{2m_{s}}\left(\Psi \nabla \Psi^{⋆}-\Psi^{⋆}\nabla \Psi \right)-\frac{q_{s}}{m_{s}}{\left|\Psi \right|}^{2}\left(q_{s}A-\alpha \right).$$

Equation (6) is obtained by taking the divergence of Equation (5). Since the external magnetic field $B_{e}=\mu_{0}H_{e}$ is already defined via its vector potential $A_{e}$, it can be inferred that $\nabla \times H_{e}=0$ in the SC. The vector potential entering Equation (4) can be written as $A=A_{s}+A_{e}$, where $A_{s}$ is the solution of Equation (5) in the absence of external currents. In Equation (4) the SOC-parameter enters the expression containing the first GL-coefficient *a* and leads to an enhanced critical temperature $T_{c}^{⋆}=T_{c}+\alpha^{2}/2m_{s}a_{0}$. A space-dependent SOC-parameter modulates the effective critical temperature $T_{c}^{⋆}$ in the same way as $\alpha^{2}$. On the other hand, α also appears in the form of a vector potential giving rise to an anomalous contribution to the supercurrent density $j_{s}$. For our calculations, the SOC-parameter is chosen such that $\alpha^{2}\left(r\right)/2m_{s}\leq 0.01\left|a\right|$ and the temperature modulation is of a minor relevance. It should be noted, however, that we are operating with a rather strong SOC parameter compared with typically employed values $\left|\alpha \right|∼0.01\hbar /\xi_{GL}$[27]. It was shown that for such a value of α the spontaneous vortex–antivortex generation is possible even in the absence of external magnetic fields [28]. In our case, the simulations did not show SOC-induced vortex–antivortex pairs, but it should be noted that we are considering a different geometry of the SC/FM bilayer and also a different spin texture. We consider Nb with the same material constants as in [29] and a normal conductivity of $\sigma =38{\left(\mu \Omega \;m\right)}^{-1}$. This yields a relaxation coefficient γ=0.016. Nb is well studied and documented. On the other hand, the combination of Nb and ${\mathrm{TbMnO}}_{3}$ is probably not optimal, since the vortex diameter in Nb is much larger than the wave length of the spin spiral, which is in the order of several nanometers. High-Tc superconductors with $\kappa \gg 1/\sqrt{2}$ offer a better match. The wave length of the spin spiral was chosen to have minimum of Λ=400 nm, which is roughly 2.5 times the vortex diameter $d_{v}=2\xi_{GL}$ in our calculations. The spin ordered part is ideally an insulator with large SOC. Alternatively, SOC can also be introduced to the system by adding a thin metallic interlayer with strong intrinsic SOC in between the bilayer [30]. To be specific, we assume a helical spin texture and an exchange field in the form of a Néel spiral

(8) $$h=-\sin\left(2\pi \frac{nx}{d}\right)e_{x}-\cos\left(2\pi \frac{nx}{d}\right)e_{z}.$$

The winding number *n* corresponds to the number of complete rotations of h from one end of the SC to the other. The absolute value of the field h is absorbed into the SOC-parameter and h is therefore a unit-vector field. With this we infer for the SOC-parameter

(9) $$\alpha =-\alpha_{0}\sin\left(2\pi \frac{nx}{d}\right)e_{y}.$$

Considering the similarity between the SOC-parameter and a vector potential, one realizes that the corresponding magnetic field points perpendicular to the interface

(10) $$B_{SOC}=-\nabla \times \frac{\alpha }{q_{s}}=2\pi n\frac{\alpha_{0}}{dq_{s}}\cos\left(2\pi \frac{nx}{d}\right)e_{z}.$$

and modulates the external magnetic field $B_{e}$. In contrast to the external field, the spin–orbit field has a spatial dependency and its magnitude increases with the winding number *n*. Since the SC is constructed such that $d\ll \xi_{GL}<\lambda_{GL}$, the dipolar fields of its Meißner currents can be neglected and Equation (5) does not need to be solved explicitly. Instead, Equations (4) and (6) are solved with the methods described in [29]. For the boundary conditions we have

(11) $$\left(\hat{D}+\alpha \right)\Psi \cdot N_{v}=0,\;\;\mathrm{on}\;\;\partial \Omega_{v}$$

(12) $$\Psi =0,\;\;\mathrm{on}\;\;\partial \Omega_{e}$$

(13) $$-\sigma \nabla \phi \cdot N_{e}=j_{e},\;\;\mathrm{on}\;\;\partial \Omega_{e}$$

$\Omega_{v}$ and $\Omega_{e}$ are the SC/vacuum and SC/electrode boundaries and $N_{v}$ and $N_{e}$ the corresponding surface normals. These boundary conditions are most commonly used for simulating current flows in SCs [31,32], but other formulations may also be employed [33]. For simplification purposes, the thickness of the FM $h_{FM}$ is chosen to be small enough that its dipolar fields can be neglected [20]. In addition, the back-action from the SC on the FM is not taken into account [34], and the FM is supposed to be unaffected by all considered magnetic fields. However, in general even weak magnetic fields can lead to a spatial distortion of the magnetic texture. The broken spatial inversion symmetry then allows for additional texture-induced Lifschitz invariants in the free energy, which should be taken into account for a more complete picture [35]. Oersted fields of the remaining parts of the circuit are neglected.

## 3. Results and Discussions

### 3.1. Vortex Dynamics in the Absence of SOC

The problem of vortex matter in the presence of transport currents has been studied for a long time and the key principles in extended systems are well understood [23,36]. The Lorentz force resulting from the transport current drives the fluxons into a state of viscous flow, with a moving direction being transverse to the current and to the applied magnetic flux. A moving vortex thereby induces an electric field in the direction of the current flow, which causes a finite electrical resistance. In clean samples with no pinning centers, the heat generation and energy loss caused by the vortex motion deteriorate the device efficiency. Therefore, much effort has been devoted to understanding how the flux-motion can be inhibited. Here we are dealing with vortex pinning caused only by electromagnetic fields and geometric confinement, since our SC is assumed to be free of defects. We note that the presence of impurities and the influence of pinning centers can considerably change the conditions for vortex entry and flux motion, as discussed in numerous studies [37,38,39].

**Figure 2:**
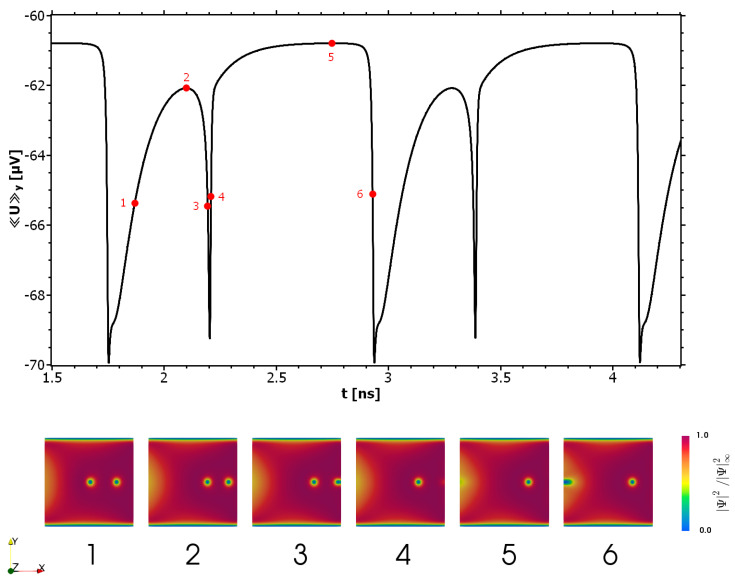
Time evolution of the SC for $B_{e}=1.5\;\mathrm{mT}$, $\alpha_{0}=0$, and $j_{e}=7.07\;\mathrm{GA}/m^{2}e_{y}$. Upper panel: mean voltage versus time over two complete periods. The numbering of the red points corresponds to that of the lower panels and marks the vortex state of the SC. Lower panel: time evolution of the vortices. The red color corresponds to the fully superconducting material and blue to the normal conducting regions.

We start studying the vortex motion by slowly ramping up the external current from an initial state with one vortex in the center of the SC. In this state we set $B_{e}=1.5\;\mathrm{mT}$ with no SOC or equivalently n=0. For small current densities $j_{e}=j_{e}e_{y}$ the vortex is pushed in the x-direction until it finds a new equilibrium position closer to the edge at x=2μm. A new equilibrium is established since vortex expulsion is prevented by a surface barrier that, in mesoscopic samples, is typically a hybrid effect of the microscopic Bean–Livingston barrier and the geometric confinement [38,40,41]. If the current reaches a critical value $j_{d}$, the vortex motion sets in and the Lorentz force becomes strong enough to push the fluxons across the barrier. In this regime a steady vortex motion is reflected in periodic oscillations of the mean voltage (see Figure 2)

(14) $${\langle U\rangle }_{x,y}=\frac{1}{d}\int_{\Omega_{SC}}\nabla \phi \cdot e_{x,y}dA.$$

When the critical current is surpassed, a flux motion is triggered by a vortex entering the sample from the left and traveling towards the center. The Lorentz force and the repulsive vortex–vortex interaction drive the fluxon pair towards the edge until an expulsion event happens. After this stage the flux motion stops for a moment and the new vortex remains static until another vortex enters the SC from the left edge. After that the whole process starts again. In general, the fluxons in mesoscopic systems are affected in a complicated way by the edge barrier, which in turn depends on the number of fluxons in the system and on their distribution. In addition, the edge imperfections and the strength of the applied magnetic fields, as well as the geometry of the SC, are important factors. A detailed analysis of these issues is beyond the scope of this work. We focus mainly on the influence of SOC on the vortex dynamics. In the following section we demonstrate how the edge barrier is affected by the magnetic texture and how the vortex motion changes in the presence of SOC.

### 3.2. Vortex Dynamics in the Presence of SOC

We start investigating the vortex dynamics in the presence of SOC by slowly ramping up the winding number from n=0 to |n|=10 in the dynamic state introduced in the last section (see Figure 2). This can be done by acting on the magnetic layer with an electric field that couples to the ferroelectric polarization associated with the spin helical ordering. For instance, when the ferroelectric polarization vanishes, we reach the ferromagnetically ordered state. In the simulation we tune the SOC-parameter such that $\left|\alpha \right|=0.1\hbar /\xi_{GL}$, whereas the values of the external magnetic field and of the current remain the same. A change in the winding number leads to a spatial variation of the magnetic field in the SC with the strength and distribution being determined by Equation (10). The gate voltage applied to the magnetically active elements is symmetric with respect to the central axis $e_{y}$ of the SC with a constant local magnetic moment and exchange field $h=-e_{z}$ along the y-axis. We do not simulate the transient spin dynamics in the magnetic layer itself. We consider two scenarios where the local magnetic moment is not fixed in the center but along one of the two edges (x=±d/2,y), yielding two additional SOC-parameters with indices *l* (left) and *r* (right) marking the edge of fixed local magnetic moments

(15) $$\alpha^{l,r}=-k_{r,l}\alpha_{0}\sin\left(n\pi \left[2\frac{x}{d}+k_{r,l}\right]\right)e_{y}$$

with $k_{l}=1$ and $k_{r}=-1$. The corresponding magnetic field is

(16) $$B_{SOC}^{l,r}=k_{r,l}2\pi n\frac{\alpha_{0}}{dq_{s}}\cos\left(n\pi \left[2\frac{x}{d}+k_{r,l}\right]\right)e_{z}$$

**Figure 3:**
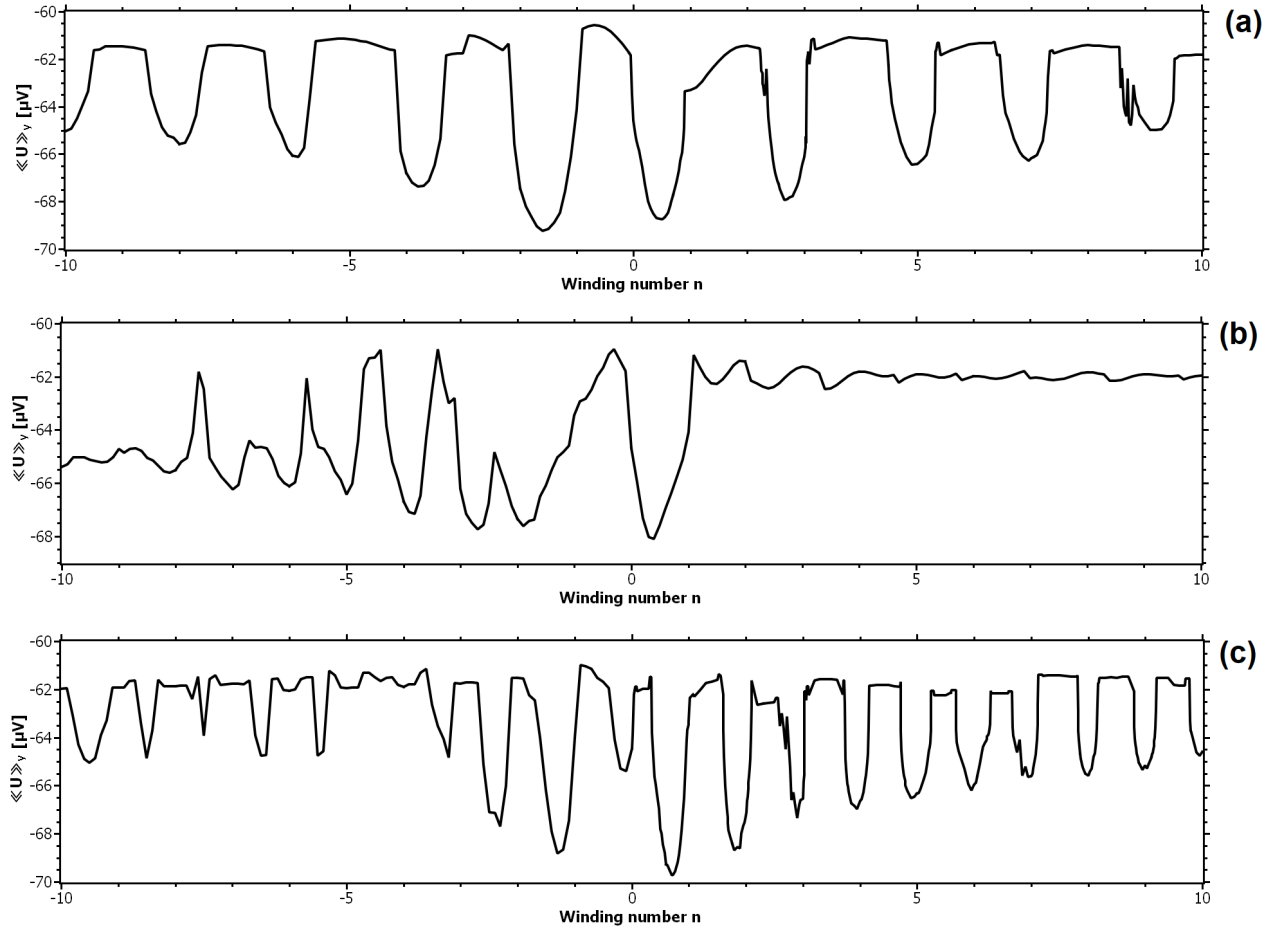
Mean voltage versus winding number for $B_{e}=1.5\;\mathrm{mT}$, $\alpha_{0}=0.1\hbar /\xi_{GL}$, and $j_{e}=7.07\;\mathrm{GA}/m^{2}e_{y}$. (a) The magnetization is symmetrically contracted around the y-axis with $\alpha =\alpha^{s}$. (b) The magnetization is fixed at x=−d/2 with $\alpha =\alpha^{l}$. (c) The magnetization is fixed at x=+d/2 with $\alpha =\alpha^{r}$.

**Figure 4:**
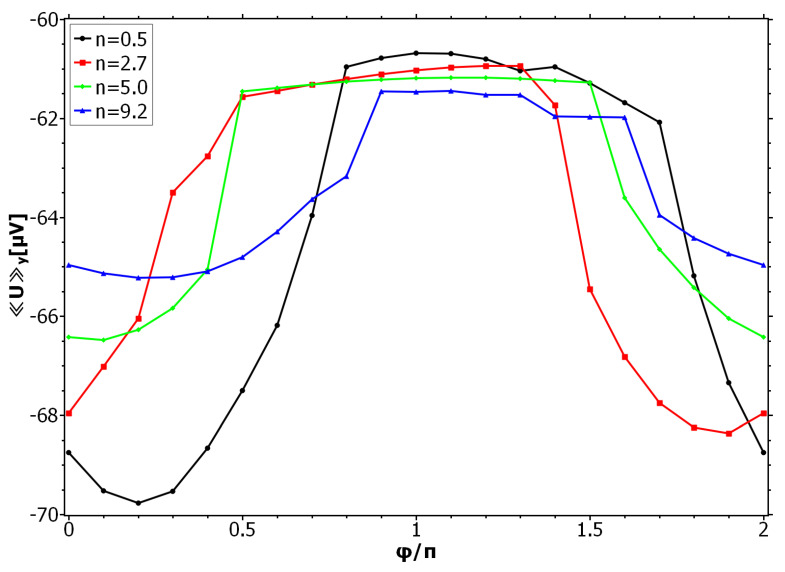
Mean voltage versus phase shift of $\alpha^{s}$ for specific winding numbers. The values of *n* are chosen such that for φ=0 we are in the resistive state (see Figure 3a). An appropriate phase shift switches the system from being resistive to being dissipationless.

For the remainder of the text, the field of symmetric magnetic moment contraction is marked as $\alpha^{s}$. For all considered values of *n*, the vortex motion is studied by calculating the space and time-averaged voltage across the SC. The effect of ramping up |n| for all three cases $\alpha =\alpha^{s,l,r}$ is shown in Figure 3. With increasing the winding number the mean voltage starts to oscillate. Each period consists of a sharp minimum of *U* followed by an interval of relatively constant voltage. Analysis of the simulation data reveals that the pronounced voltage minima are due to a greater vortex mobility, which is enabled by an easier vortex penetration into the sample. The plateaus of the constant voltage are due to vortex pinning, which is caused by impeded vortex penetration. In all calculations we observed that the entry into the resistive state was triggered by a vortex entering the sample, and therefore the edge barrier for vortex penetration is crucial for dynamics. The barrier for vortex exit is of lesser relevance. This observation is also reflected by the voltage curves in Figure 3b,c, where the SOC field has a fixed value at one of the edges. In Figure 3b, the magnetization is fixed at the left edge of the sample where vortex penetration happens. For negative values of *n*, $B_{SOC}$ has a minimum, whereas for positive *n* it is maximal. For a positive SOC field at the left edge, the vortex motion is permanently inhibited, whereas for negative values sharp voltage oscillations appear with a higher frequency than in the previous case. This observation suggests that the edge barrier for vortex penetration depends on the sign and strength of $B_{SOC}$ and can be controlled with an appropriate phase shift of the spin spiral. Such a phase shift can indeed be utilized to trigger vortex pinning and switch the system from the resistive to the dissipationless state. To demonstrate this phenomenon we modified the SOC-parameter $\alpha^{s}$ according to

(17) $$\alpha^{s}=-\alpha_{0}\sin\left(2\pi \frac{nx}{d}+\varphi \right)e_{y}$$

with a phase factor φ∈[0,2π] that was ramped up for specific n-values of the curve of Figure 3a. The phase shift was performed for values of *n* for which the vortex motion is enhanced and the mean voltage has a minimum. The results are presented in Figure 4. The vortex pinning can be restored by shifting φ by π, which goes hand in hand with a sign inversion of $B_{SOC}$. As for $\alpha =\alpha^{l}$ and for fixed magnetic ordering at the left edge, also for the symmetric field $\alpha^{s}$, the vortex motion was observed to be enhanced by negative values of $B_{SOC}$ around the left edge of the SC. The phase shift of φ restores the pinning by switching the sign of the SOC field back to positive, thereby increasing the edge barrier. In the calculations for Figure 3c, the field at the left edge was variable, whereas the field on the right edge was set to have a positive value for negative *n*, and a negative value for positive *n*. In line with previous observations, the frequency of the oscillations has roughly doubled since $B_{SOC}$ also changes sign twice as fast at the left edge of the SC for $\alpha =\alpha^{r}$ with increasing *n*. The asymmetry in the curve suggests that the vortex expulsion barrier, despite its lesser relevance in the given problem, plays a role in the vortex dynamics.

**Figure 5:**
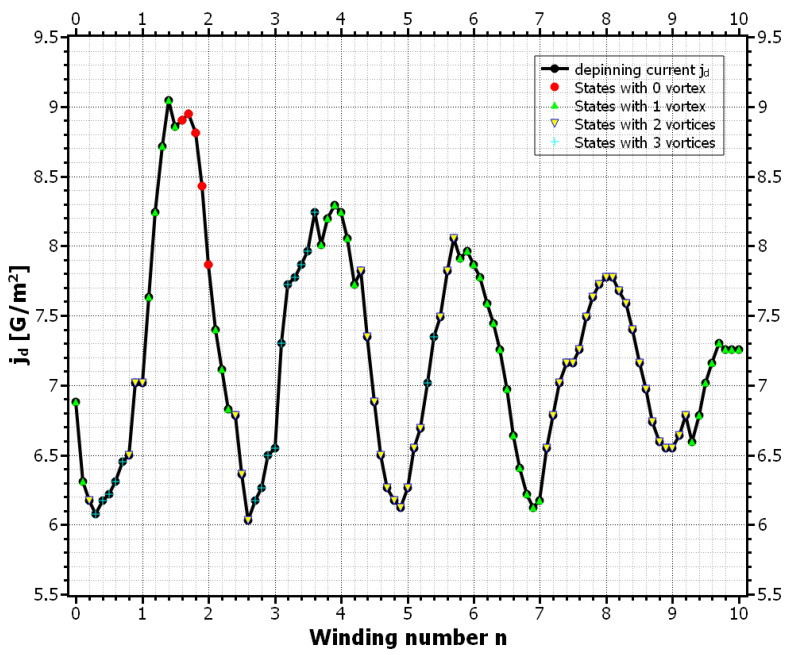
Depinning current versus the winding number for $B_{e}=1.5\;\mathrm{mT}$, $\alpha =\alpha^{s}$, and $\alpha_{0}=0.1\hbar /\xi_{GL}$. For $j_{e}\leq j_{d}$ the vortex motion is prevented, whereas for $j_{e}>j_{d}$ the SC enters the resistive state. The shape of the approximate curve also depends on the number of vortices and has kinks where this number changes.

**Figure 6:**
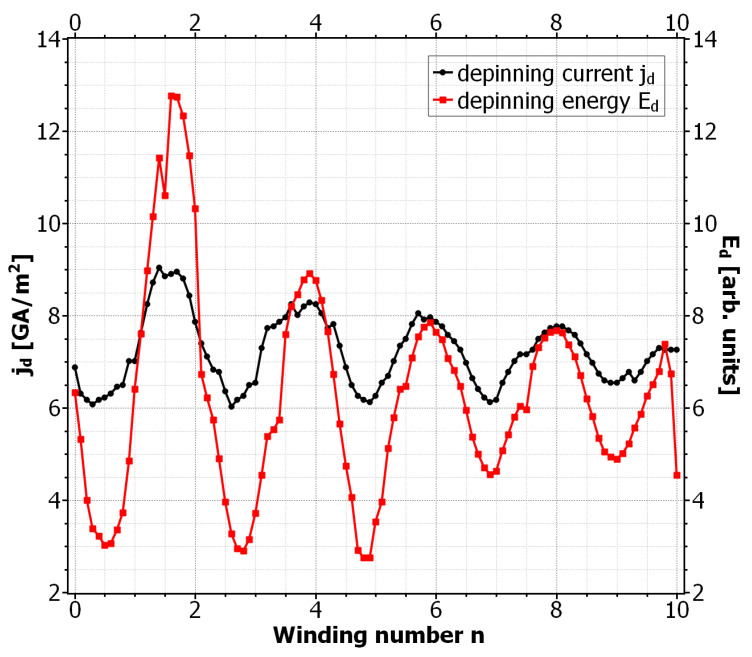
Depinning current and corresponding energy for $B_{e}=1.5\;\mathrm{mT}$, $\alpha =\alpha^{s}$, $\alpha_{0}=0.1\hbar /\xi_{GL}$, and $j_{e}=7.07\;\mathrm{GA}/m^{2}e_{y}$. The depinning energy $E_{d}$ corresponds to the energy barrier for the vortex entry since the permanent vortex motion was always observed to be triggered by a vortex entry event.

**Figure 7:**
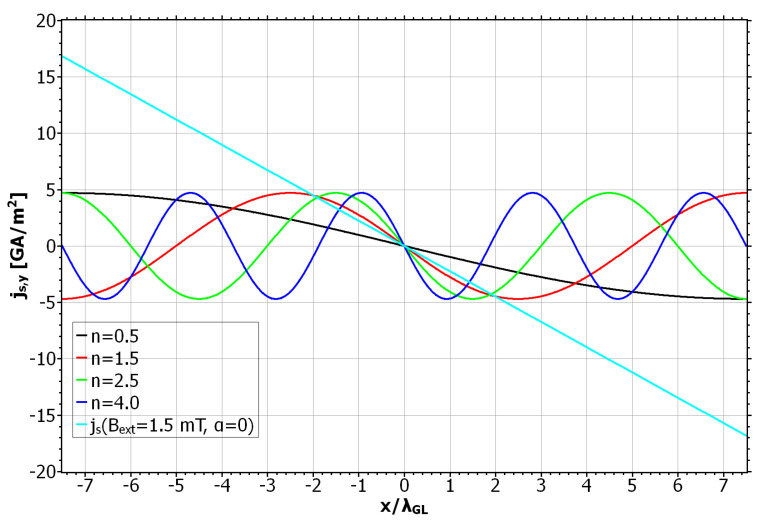
Supercurrent distribution of a reference state with $\alpha =\alpha^{s}$ and without vortex. y-component of the supercurrent along the x-axis for values of *n* for which the depinning current becomes extremal. Pinning is observed to happen when the spin–orbit field becomes maximal around the left edge $x=-7.5\lambda_{GL}$, whereas the flux motion is enhanced for negative values.

The complete phase diagram of static and dynamic vortex phases due to the SOC field $\alpha^{s}$ is shown in Figure 5. Here the black curve approximates the critical current densities $j_{d}$ for which the vortex motion is barely inhibited. As in previous calculations, the depinning current oscillates with the winding number. For large winding numbers, the maxima and minima of $j_{d}$ coincide with the even and odd values of *n*, respectively. For small winding numbers these extrema are shifted to half integer values. Another interesting observation is that the vortex number along the curve changes more rapidly for small values of *n*, whereas for large winding numbers only 1-vortex and 2-vortex states appear. The maximum depinning current appears at around n=1.5 and has a roughly 28% higher value than in the SOC-free case. In Figure 6 we plotted the critical energy for the vortex penetration $E_{d}$, together with the corresponding depinning current $j_{d}$. We can see that the maxima and minima of the curves coincide very well and therefore again the modified penetration barrier at the left edge is identified as the cause for the alternating depinning current. For a better understanding of the high and low *n* regimes in the curves, we plotted the screening current distribution $j_{s,y}\left(x,y=0\right)$ due to the SOC field for different values of the winding number (see Figure 7 ). The reference state with no vortex and $B_{ext}=0$ and $j_{ext}=0$ was used for this calculation, which means the screening currents are purely determined by the anomalous part of $j_{s}$, which for |Ψ|≈1 is given by $j_{s}=q_{s}/m_{s}\alpha^{s}$. For comparison, the usual screening current due to $B_{ext}$ and with α=0 was also plotted. We can see that for arbitrary *n* the SOC-induced current distributions are equal in magnitude. This is because the supercurrents always encircle the same magnetic flux over one period of $B_{SOC}$, regardless of winding number and the field strength of $B_{SOC}$. For small winding numbers the screening currents are broadly distributed. As we saw earlier, the vortex penetration is preferred here for n=0.5 and n=2.5. For these values the screening currents along the left edge due to α and $A_{ext}$ are maximal and add up. The effective screening current is therefore enhanced and leads to a stronger suppression of |Ψ| and a stronger supervelocity along the edge. This in return favors the vortex entry and reduces the corresponding edge barrier [37]. If the currents oppose each other, like for n=1.5, the total current distribution is reduced. This situation is similar to that of a recovery of the edge barrier due to vortex entry [42]. In our situation it has the same effect and prevents the vortex entry, which in return keeps the already existing vortices static. For larger values of *n*, the maxima and minima of the screening currents are no longer located at the edges of the SC when $j_{d}$ becomes extremal. Instead, the corresponding currents now flow inside the SC and are zero at the edges. However, the effects that determined the vortex mobility for small *n* are still active and one can see that vortex pinning still happens when the total current around the left edge is reduced. So we conclude that it is not the value of the supercurrent but rather its total distribution around the edges that determines the vortex velocity in mesoscopic systems. This observation is supported by the fact that for broader current distributions, apparently more vortices are able to simultaneously exist inside the SC leading to a stronger voltage signal and a reduced $j_{d}$.

**Figure 8:**
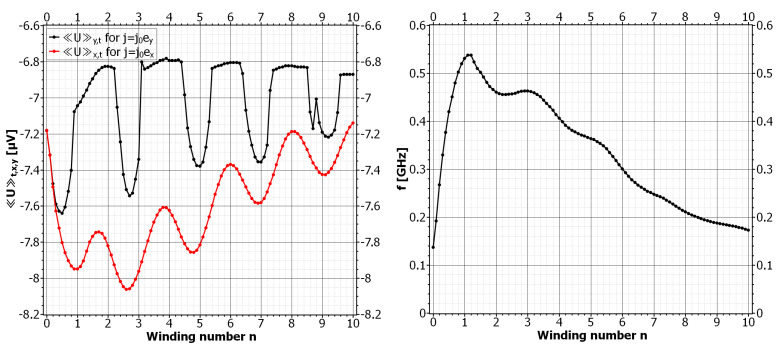
Left: Mean voltage versus winding number for $\alpha =\alpha^{s}$ and a transport current in the y-direction (upper curve) and x-direction (lower curve). For $j_{e}=j_{e}e_{y}$ vortices are forced to move along k and experience subsequent phases of pinning and motion depending on *n*. For $j_{e}=j_{e}e_{x}$ vortices move transverse to k and no pinning is observed for positive winding numbers. Right: Frequency of the voltage oscillations for $j_{e}=j_{e}e_{x}$ depending on the average vortex number and the time a vortex resides in the sample.

The transport current can proceed along the x-direction and transverse to $\alpha^{s}$. The vortex motion is investigated by calculating the mean voltage for different values of *n*. For a comparison, the mean voltage for the case $j_{e}=j_{e}e_{y}$ is plotted (see Figure 8). The voltage oscillates with the winding number but there is no regime of vortex pinning for positive *n*. Instead, the voltage signal is strongly enhanced, which is most pronounced for small winding numbers. Inspection of the simulation data revealed that a higher average number of vortices in the SC is the reason for this enhancement. The average vortex number is continuously changing with *n*, and has maxima and minima that coincide with the voltage extrema for the case $j_{e}=j_{e}e_{x}$. The reason for the oscillating mean voltage is still not fully understood, since the complicated boundary-current distribution due to $\alpha^{s}$ has so far prevented a reliable conclusion. However, vortex entry was again observed to take place where $B_{SOC}$ has a maximum at the lower boundary. For the symmetric SOC parameter, the SOC field is always positive at the center of the SC and builds a channel that vortices cannot leave in the direction transverse to α. Only for small *n* does the broad distribution of $B_{SOC}$ allow for lateral fluxon movement. For n>1.3, the fluxon motion was observed to be highly directional along $e_{y}$. We recorded the frequency of the time-dependent voltage oscillations that depends on the average vortex number, the vortex velocity, and the time period a vortex resides in the SC before it is expelled. The frequency of the oscillations are in the GHz regime with a maximum at n=1. For this particular value we observed the highest number of vortices simultaneously moving in the SC. For larger winding numbers the frequency decreases and approaches the initial value for $B_{SOC}=0$. The out-averaging of the SOC field is observable in the voltage curves where it manifested in decreased amplitudes of the voltage minima. For large winding numbers the amplitudes of $j_{d}$ are damped. Allowing for negative values of *n* (not shown here), the mechanism that enhances the vortex–vortex mobility is reversed, and we enter the regimes of vortex pinning with a constant mean voltage.

## 4. Conclusions

We investigated how the superconducting vortex dynamics are affected by the proximity to a magnetically ordered top layer with strong SOC due to intrinsic helical order. As a specific example for the magnetic ordering, we considered a Néel spiral spin-texture and found the winding number to be the main control parameter for vortex motion. We inferred that by increasing the winding number, vortices experience alternating phases of enhanced mobility and pinning. We traced back this behavior to a modification of the barrier for vortex entry by the SOC-induced screening currents along the edges of the SC. Furthermore, we demonstrated that the average vortex number in the SC and the critical depinning current are also modified by the magnetic spin spiral, and that pinning is most effective for low winding numbers. For high winding numbers the SOC effect averages out and the amplitudes of $j_{d}$ tend to zero. Our observations of a modified edge barrier are supported by the fact that the vortex mobility is sensitive to the phase of the magnetic spiral at the edge where the vortex penetration happens. We also demonstrated that due to the symmetry of the SOC field, the fluxon transport in the SC is anisotropic, enabling highly channeled vortex motion for a transport current transverse to the spin spiral. The presented results point to an all-electric control of superconducting transport properties by exploiting a spin-current-induced ferroelectricity in the top layer, which renders a coupling of the spin ordering to electric fields. We also expect the results to be valid for a single planar superconductor subjected to magnetic stray fields with a distribution that resembles that of the SOC field. Investigations in this direction are ongoing. Other issues to be investigated are further facets of the Rashba-SOC, which depends on the potential gradient at the interface. Therefore, its amplitude may be influenced by the strength of the ferroelectric polarization. The latter depends linearly on the magnetic spiral winding number. Thus, the Rashba-type effective magnetic field contains a contribution that is an even function of the winding number. This secondary effect is currently being quantified by elaborate simulations.

## Data Availability

All data in this work are available from the authors upon reasonable request.
